# Sex Differences in Number Magnitude Processing Strategies Are Mediated by Spatial Navigation Strategies: Evidence From the Unit-Decade Compatibility Effect

**DOI:** 10.3389/fpsyg.2019.00229

**Published:** 2019-02-12

**Authors:** Belinda Pletzer, TiAnni Harris, Andrea Scheuringer

**Affiliations:** ^1^Department of Psychology, University of Salzburg, Salzburg, Austria; ^2^Centre for Cognitive Neuroscience, University of Salzburg, Salzburg, Austria

**Keywords:** sex differences, number magnitude processing, number comparison, unit-decade compatibility effect, holistic processing, decomposed processing, hybrid model

## Abstract

The hybrid model of number magnitude processing suggests that multi-digit numbers are simultaneously processed holistically (whole number magnitudes) and in a decomposed manner (digit magnitudes). Thus, individual tendencies and situational factors may affect which type of processing becomes dominant in a certain individual in a given situation. The unit-decade compatibility effect has been described as indicative of stronger decomposed number processing. This effect occurs during the comparison of two-digit numbers. Compatible items in which the larger number contains the larger unit digit are easier to solve than incompatible items in which the larger number contains the smaller unit digit. We have previously described women show a larger compatibility effect than men. Furthermore, the compatibility effect is modulated by situational factors like the vertical spacing of the presented numbers. However, it has not been addressed whether situational factors and sex affect the unit-decade compatibility effect interactively. We have also demonstrated that the unit-decade compatibility effects relates to global-local processing, which in turn also affects spatial processing strategies. However, a link between spatial processing strategies and the unit-decade compatibility effect has not yet been established. In the present study we investigate, whether sex differences in the unit-decade compatibility effect (i) depend on the vertical spacing between numbers, (ii) are mediated via sex hormone levels of participants, and (iii) relate to sex differences in spatial processing strategies. 42 men and 41 women completed a two-digit number comparison task as well as a spatial navigation task. The number comparison task modulates compatibility and vertical spacing in a 2 × 2 design. The results confirm a larger compatibility effect in women compared to men and with dense compared to sparse spacing. However, no interactive effect was observed, suggesting that these factors modulate number magnitude processing independently. The progesterone/testosterone ratio was related to the compatibility effect, but did not mediate the sex difference in the compatibility effect. Furthermore, spatial processing strategies were related to the compatibility effect and did mediate the sex difference in the compatibility effect. Participants with a stronger focus on landmarks in the spatial navigation task showed a larger compatibility effect.

## Introduction

Number magnitude processing has been extensively studied using various versions of number comparison tasks (for reviews see Dehaene et al., 2003; Ballan, 2012). Tasks differ in whether the numbers to be compared are single or multi-digit numbers, whether numbers are compared to a fixed standard or variable and – relatedly – whether they are presented simultaneously or consecutively (Ballan, 2012). Using any of these versions, it has been well established that the comparison of two numbers becomes harder, the larger they are (***problem size effect;*** for a review see Ashcraft and Guillaume, 2009) and the smaller the numerical distance between them (***distance effect;*** Dehaene, 1989; Reynvoet and Brysbaert, 1999; Nuerk et al., 2001; Huber et al., 2017). A common model of number magnitude processing is the mental number line (e.g., Wood and Fischer, 2008). This mental number line is assumed to be logarithmically compressed (e.g., Dehaene, 2003; Dehaene et al., 2008). That means that smaller numbers, which people use more frequently, have more distinct representations on the mental number line than larger numbers, which are less frequently used (e.g., Dehaene, 2003; Dehaene et al., 2008). Number comparison tasks can be understood as judging the relative positions of two numbers on the mental number line. The logarithmic compression of the mental number line can explain the problem size effect. Furthermore, if the relative positions of the two numbers on the mental number line are closer together, they are harder to distinguish, which results in the distance effect (e.g., Verguts and Van Opstal, 2005). The mental number line represents a spatial representation of numbers. Indeed number magnitude processing shares multiple features with spatial processing (Hubbard et al., 2005). Spatial and numerical processing interfere in various tasks and share common neural substrates (Hubbard et al., 2005).

There is accumulating evidence that spatial processing is influenced by basic visual attentional processes, like global-local processing (see e.g., Handa and McGivern, 2015 for a review). Most stimuli in everyday life are hierarchical with global patterns made up of local parts (Navon, 1977). When a visual stimulus is encountered, both the global pattern and its parts are processed simultaneously (see Kimchi, 1992 for a review). Usually global processing occurs faster than local processing (***global advantage effect***), but situational and individual characteristics affect performance in global-local processing tasks (e.g., Martin, 1979; Navon and Norman, 1983; Fink et al., 1997; Roalf et al., 2006; Razumnikova, 2011). Importantly, many studies find a global processing tendency in men, but local processing tendency in women, as indicated by a larger global advantage effect in men compared to women (Roalf et al., 2006; Razumnikova, 2011; Pletzer et al., 2014; Scheuringer and Pletzer, 2016; but see Pletzer and Harris, 2018). These differences were modulated by women’s menstrual cycle phase and we were able to link sex differences in global-local processing to sex hormone influences. While testosterone relates to an increased global advantage effect (Pletzer et al., 2014), progesterone related to a decreased global advantage effect (Pletzer et al., 2014; Pletzer and Harris, 2018). Accordingly, sex differences were largest, when women were in their luteal cycle phase, i.e., when their progesterone levels peak (Pletzer et al., 2014).

These differences in global-local processing also influence spatial processing (Basso and Lowery, 2004; Pletzer et al., 2017). Multiple studies demonstrate that men outperform women in spatial tasks like spatial navigation or mental rotation (see Andreano and Cahill for a review), while a female advantage has been observed in other spatial processing tasks like e.g., object location memory (see Voyer et al., 2007 for a meta-analysis). Sex differences in spatial navigation and mental rotation are robust, as demonstrated by meta-analyses (Voyer et al., 1995 for a meta-analysis) and cross-cultural studies (Silverman et al., 2007; Janssen and Geiser, 2012). These sex differences have in part been attributed to the use of different processing styles in men and women (see Pletzer, 2014 for a review). For instance during mental rotation, men seem to use a Gestalt-approach and rotate the stimuli holistically, while women seem to use a more detail-oriented approach and rotate stimulus parts (Gluck and Fitting, 2003; Peña et al., 2008; Rilea, 2008; Janssen and Geiser, 2012). Likewise, during spatial navigation, men seem to take an allocentric perspective and use an Euclidian representation of the environment (Euclidian strategy), while women seem to take an egocentric perspective and use landmarks in the environment (landmark-based strategy) for navigation (Galea and Kimura, 1993; Dabbs et al., 1998; Lawton, 2001; Saucier et al., 2002; Andersen et al., 2012; Harris et al., unpublished). Furthermore, the use of landmark-based strategies during navigation increases in the high progesterone luteal phase of the menstrual cycle (Hussain et al., 2016; Scheuringer and Pletzer, 2017).

A more holistic processing style during spatial tasks has repeatedly been linked to global processing (Basso and Lowery, 2004; Pletzer et al., 2017). For instance a larger global advantage effect during global-local processing tasks predicts the use of an allocentric perspective during spatial navigation (Pletzer et al., 2017). It has also been demonstrated that a more holistic processing style, is beneficial for performance in spatial tasks (Dabbs et al., 1998; Saucier et al., 2002; Janssen and Geiser, 2012). For example, allocentric perspective use during navigation reduces errors in a mental rotation task (Saucier et al., 2002).

Like spatial stimuli, numerical stimuli are hierarchical with multi-digit numbers being composed of single digits. Accordingly, the question arises, whether global-local processing also transcends to cognitive processing in the numerical domain. Independent of the global-local processing literature, it has been discussed, whether number comparison occurs holistically, i.e., by comparing whole number magnitudes, or in a decomposed manner, i.e., by comparing units, decades, hundreds, etc. separately. The holistic model assumes a single logarithmically compressed mental number line along which whole number magnitudes are represented (Dehaene et al., 1990; Brysbaert, 1995). The decomposed model assumes decade breaks in the mental number line, resulting in separate representation of unit, decade and hundred magnitudes (Nuerk et al., 2001).

Evidence for the decomposed model comes from the so called ***unit-decade compatibility effect***, which occurs when comparing variable pairs of two-digit numbers (Nuerk et al., 2001). Items are referred to as compatible, if the larger number contains the larger unit digit (e.g., 69 vs. 21; 6 > 2, 9 > 1), but as incompatible, if the larger number contains the smaller unit digit (e.g., 61 vs. 29; 6 > 2, 1 < 9). It has repeatedly been demonstrated that incompatible items are more error prone and solved more slowly than compatible items (unit-decade compatibility effect).

More recent models of multi-digit number processing (hybrid model) assume that holistic and decomposed number processing occur in parallel. Accordingly, a stronger unit-decade compatibility effect can be viewed as indicative of stronger decomposed number processing. This further outlines similarities between number magnitude processing and basic global-local processing of visual stimuli. Indeed we were recently able to link the unit-decade compatibility effect during number comparison to the global advantage effect in a global-local processing task (Pletzer et al., unpublished). The smaller the global advantage effect, the larger is the unit-decade compatibility effect. Accordingly, it has been established, that both spatial and numerical processing are influenced by basic visual attentional processes. It is therefore reasonable to assume that factors affecting global-local processing may transcend into the spatial and numerical domain.

Multiple studies have now demonstrated sex differences in the unit-decade compatibility effect (Pletzer et al., 2013; Harris et al., 2018), including a large-scale online study (Huber et al., 2017). Women show a larger compatibility effect in reaction times (RT) than men. Huber et al. (2017) further demonstrate that among several individual characteristics, sex is the only factor to significantly affect the compatibility effect in RT, even after controlling for other factors relevant to numerical cognition, such as age, education, math grades. In addition Huber et al. (2017) also found a trend toward sex differences in the compatibility effect in ER, with women showing a larger compatibility effect in ER than men. Furthermore, previous studies demonstrate, that behavioral sex differences in the compatibility effect are accompanied by sex differences in brain activation patterns (Pletzer et al., 2013). Taken together, these data suggests that on average men show a higher tendency toward holistic processing, but women on average show a higher tendency toward decomposed processing. The fact that men and women differ in their tendency to process numbers holistically or in a decomposed fashion supports the notion of individual differences in the tendency to process numbers holistically, which is in line with the hybrid model of number magnitude processing (Verguts and De Moor, 2005). Accordingly, individual tendencies and situational factors may affect which type of processing (holistic or decomposed) becomes dominant in a certain individual in a given situation.

In a more recent behavioral study however, we failed to replicate the sex difference in the compatibility effect when numbers where presented to the centre of the screen like in the previous fMRI study (Harris et al., 2018). The sex difference was only confirmed when numbers were presented to the left or right hemifield (Harris et al., 2018). We speculated that these inconsistencies between the two studies may be the result of differences in the way stimuli were presented. Due to different screen resolutions, the vertical spacing between the two numbers to be compared was larger in the scanner environment (Pletzer et al., 2013) than in the behavioral lab (Harris et al., 2018). Thus, the fact that a sex difference was observed in one study, but not in the other, may result from an interaction between individual processing tendencies and situational aspects.

Indeed we were able to demonstrate in a sample of men that the size of the compatibility effect depends on presentation mode (Pletzer et al., 2016). For instance, the compatibility effect was only observed, when numbers were presented simultaneously on the screen as compared to consecutive presentation, suggesting that a decomposition of multi-digit numbers into units and decades is facilitated by simultaneous processing. Furthermore, we demonstrated that the compatibility effect is reduced with larger vertical spacing between numbers, suggesting that this facilitation of decomposed processing by simultaneous presentation is diminished if numbers are not presented close enough to each other to allow for efficient simultaneous processing. If this is the case, a larger vertical spacing may enhance the individual tendency to process holistically in men, which could explain the inconsistencies between our previous studies.

Accordingly, it has been established that like spatial processing and global-local processing, number magnitude processing is influenced by task characteristics and individual characteristics, like participant’s sex. However, so far sex differences and task characteristics have been addressed in separation and it has not been established whether they interactively modulate number processing. Furthermore, not all factors affecting global-local processing have also been investigated in the numerical domain. For instance no study has yet addressed whether sex hormones relate to the compatibility effect during number comparison. Finally, a pattern has emerged, suggesting that sex differences in basic global-local processing may transcend into the spatial and numerical domain, manifesting in sex differences in spatial and numerical processing styles. However, a link between spatial and numerical processing styles remains yet to be established.

To investigate these questions we employed the two-digit number comparison task in a sample of healthy young men and women, while varying the vertical spacing between the two numbers as in Pletzer et al. (2016). As observed in previous studies, we hypothesize a larger compatibility effect in women compared to men and with dense vertical spacing compared to sparse vertical spacing. Moreover we hypothesize a significant interaction between sex and vertical spacing in such a way that the sex difference in the compatibility effect is larger with larger vertical spacing between the numbers. Furthermore, all women were tested in their luteal cycle phase, when progesterone levels peak, and sex hormone levels were assessed from saliva samples in all participants. We hypothesize a larger compatibility effect in participants with higher progesterone and lower testosterone levels. Finally, the same sample also completed a spatial navigation task, for which results have been summarized in Harris et al. (unpublished). Since spatial and numerical processing styles are equally influenced by global-local processing (Basso and Lowery, 2004; Pletzer et al., 2017; Pletzer et al., unpublished), we hypothesize that participants with a stronger focus on landmarks in the spatial navigation task, also show a larger compatibility effect.

## Materials and Methods

### Participants

Forty-three healthy young men and 44 healthy young women between the ages of 18 and 35 years participated in this study. According to self-reports, all participants were right-handed, had no psychological, endocrinological, or neurological disorder and were free of medication. In order to adequately assess hormonal influences on the number comparison task, only women who did not take hormonal contraceptives, and had a regular menstrual cycle between 21 and 35 days of length were allowed to participate. Furthermore, all women were tested in their mid-luteal cycle phase, 3–11 days after ovulation, i.e., 11–3 days before the onset of next menses. Ovulation was confirmed by commercial ovulation tests and onset of next menses was evaluated in follow-up. Furthermore, cycle phase was confirmed by hormone analyses as described below. Three women were excluded due to low progesterone as a result of early onset of their next menses. The remaining women had a mean cycle length of 29.29 days (*SD* = 2.82 days) and were on average tested on day 22.28 (*SD* = 3.5 days). Furthermore one men displayed extremely high estradiol values and were thus excluded from the analyses. The remaining sample consisted of 42 healthy young men (mean age: 24.28 years, *SD* = 2.39 years) and 41 healthy young women (mean age: 23.58 years, *SD* = 3.60 years). To ensure that any sex differences observed cannot be attributed to age, education or IQ, these variables were matched between groups. All participants had completed a minimum of 8 years of higher education and had passed the general qualification for university entrance. Age did not differ significantly between men and women [*t*_(81)_ = 1.05, *p* = 0.29]. Furthermore, men and women did not differ in their IQ as assessed with the Advanced Progressive Matrices (APM) Screening implemented in the Vienna Test System (WTS) [men: 108.35, *SD* = 9.18; women: 107.56, *SD* = 13.48; *t*_(81)_ = 0.32, *p* = 0.75].

### Ethics Statement

All participants gave their informed written consent to participate in the study. All methods conform to the Code of Ethics of the World Medical Association (Declaration of Helsinki). According to §163 (1) of the institutional guidelines of the University of Salzburg^1^ it is necessary to seek ethical approval for research on human subjects if the study affects their physical or psychological integrity, their right for privacy or other important rights or interests of the subjects or their dependents. Paragraph §163 (2) states that it is the decision and responsibility of the PI to decide, whether (1) applies to a study or not. These guidelines are in accordance with national regulations. This study uses only non-invasive methods on healthy adult volunteers, who volunteered to participate in the study and all data was processed in anonymized/de-identified form. Accordingly, (1) did not apply and we did not seek ethical approval for this study.

### Procedure

Participants completed (i) an attention task (not described in this manuscript), (ii) a two-digit number comparison task, and (iii) a spatial navigation task. This manuscript focuses on the results of the two-digit number comparison task and it’s relation to the spatial navigation task. Sex differences in the spatial navigation task are described elsewhere (Harris et al., unpublished). Upon arrival at the lab, participants were asked to rinse their mouth to remove particles before saliva sampling. They then completed the written consent form, as well as a general screening questionnaire to ensure they fulfilled all inclusion criteria. Tasks were performed in the order described. The first saliva sample was taken before the first task, the second saliva sample after the number comparison task and the third sample after the spatial navigation task. The IQ screening was performed as last measure of the study. The total session lasted for 1.5 h.

### Number Comparison Task

As part of a larger study, participants completed a two-digit number comparison task as described in Pletzer et al. (2016). The task was adapted from Nuerk et al. (2001). In each item, two two-digit numbers were presented vertically above each other and participants had to decide as quickly as possible which number was larger by pressing the left or right mouse key. The numbers to be compared ranged from 21 to 89 and each item included four different digits. In order to address, whether task factors, like the vertical spacing between numbers, affect sex differences in the unit-decade compatibility effect, the task varied compatibility as well as vertical spacing between numbers in a 2 × 2 design. The task was comprised of a total of 200 items, in half of which the two numbers were spaced closely together (visual angle: 11.5°), while in the other half, the two numbers were spaced farther apart (visual angle: 22°). Among the 100 items each with dense and sparse spacing, were 20 so called within-decade items, 40 compatible, and 40 incompatible items. In within-decade items the two numbers contain the same decade digit, such that units have to be compared. They were included to enhance the processing of unit-digits and avoid strategies based solely on decade digits (Nuerk and Willmes, 2005). In compatible items the larger number contains the larger unit digit (e.g., 67_43), while in incompatible items the larger number contains the smaller unit digit (e.g., 63_47). Half of the compatible and incompatible items had a small decade distance (<4), while the other half had a large decade distance. Problem size, unit distance, overall distance, and parity were matched between stimulus categories as described in Pletzer et al. (2016). The order of stimulus categories was completely randomized. Each item was presented for a maximum of 3 s until participants responded, followed by a 1.5 s inter-stimulus interval during which a fixation cross was presented. Reaction times and error rates (ER) were recorded.

### Spatial Navigation Task

In order to address, whether sex differences in the unit-decade compatibility effect relate to sex differences in spatial processing strategies.

The spatial navigation task was described in detail in Harris et al. (unpublished). In short, 20 3D navigation items were created using Unreal Engine 4 Version 8.1. The task builds upon a previous 2D-version employed by Scheuringer and Pletzer (2017), which was adapted from Saucier et al. (2002). Each item comprises a new virtual environment in which real-life landmarks (tree, bridge, stairs, and house, etc.) are randomly placed on a field representing a 10 × 10 matrix. At the beginning of each level participants were positioned on a starting field facing the new environment. After a countdown, they were informed which cardinal direction they were currently facing (north, south, east, or west) and then received directions to a target location. Their task was to reach the target location as fast as possible. Perspective and strategy were modulated in a 2 × 2 design by different phrasings of the direction: *allocentric euclidian* (“go east for 4 blocks”), *allocentric landmark* (“go east until you reach the tree”), *egocentric euclidian* (“turn right and go for 4 blocks”), and *egocentric landmark* (“turn right and go until you reach the tree”). Accordingly, landmark items referred to landmarks in the environment (“go till you reach the tree”), while Euclidian items referred to absolute distances (“go for 4 blocks”). The first four items were training items. After that participants completed 16 items, i.e., 4 per condition, in pseudo-randomized order. The time to reach the target location (navigation time) was recorded for each item.

### Hormone Analyses

After the test session, saliva samples were immediately frozen at −20°C until further analysis. Before analysis they were centrifuged at 3000 rpm for 15 and 10 min, respectively to remove solid particles. Prior to analysis the three samples were pooled to assess an average hormone value across the whole test session. Estradiol, progesterone and testosterone were analyzed from the pooled samples using DeMediTec salivary ELISA kits. For each sample, duplicate values were assessed and the average of the duplicate values was accepted if the coefficient of variation between duplicate values was below 25%. In 4 participants (1 woman, 3 men) hormone values could not be assessed due to visible blood contamination of the samples or insufficient sample volume.

### Statistical Analyses

Statistical analyses were performed in R 3.4.0. As a preparatory step, the compatibility effect was calculated for the number comparison task as mean difference between the RT/ER of incompatible and compatible items. Furthermore, for the navigation task, the strategy effect was calculated as the mean difference between landmark trials and Euclidian trials. Accordingly, a smaller strategy effect represents better performance with landmark trials. The compatibility effects in RT and ER were then analyzed in the context of linear mixed effects models using the *lmer* function of the *lme4* package (Bates et al., 2015). All models are described in detail in the respective paragraphs of the results section. All models included participant number as a random factor to control for repeated measurements. The first step was to address, whether sex and spacing affected overall performance, as well as the compatibility effect in RT and ER, sex and spacing, as well as their interaction were entered as independent variables in the lme. Age and IQ were included as covariates.

If a significant sex difference was identified, the second step was to address whether sex hormones, particularly progesterone, and testosterone related to the compatibility effect. Thus, hormone values were entered as independent variables in the linear mixed model. Since we hypothesized a positive association to progesterone, but negative association to testosterone, the progesterone/testosterone ratio was also considered as predictor of interest. For hormones that emerged as significant predictors, we assessed whether their influence mediated sex differences in the compatibility effect. Mediation analyses were performed using the *mediate* function of the *mediation* package (Tingley et al., 2014). Mediation analyses were only performed on a dependent variable, if a significant sex difference.

The third step was to address whether the compatibility effect was related to navigation strategies. Accordingly, the strategy effect in navigation as well as it’s interaction with sex were entered as predictors in the linear mixed model including also sex and it’s interaction with strategy.

In all models, non-significant interactions were backward eliminated using the *step* function implemented in the *lmerTest* package (Kuznetsova et al., 2017). Continuous dependent and independent variables were scaled prior to analyses to ensure that the b-values represent effect sizes based on standard deviations (similar to Cohen’s d).

## Results

### Effect of Sex and Spacing on Overall Number Comparison Performance

For both, RT and ER, linear mixed models were performed including participant number as a random factor and spacing (dense vs. sparse), as well as sex (men vs. women) and their interactions as fixed effects. Age and IQ were entered as covariates. Neither age nor IQ had a significant effect on RT or ER. These variables were thus removed from the models. Table 1 summarizes the respective zero-order correlations.

**Table 1:** Zero-order correlation table.

	meanRT	meanER	Comp_effect_RT	Comp_effect_ER
Age	0.02	−0.21	−0.17	−0.12
IQ	−0.13	−0.17	−0.07	−0.17
Estradiol	0.17	0.10	0.00	0.01
Progesterone	0.23^∗^	0.23^∗^	0.07	0.18
Testosterone	−0.11	−0.10	−0.06	−0.16
Progesterone/Testosterone	0.19	0.21	0.02	0.26^∗^

*Comp, compatibility; ^∗^p < 0.05.*

#### Reaction Times

**FIGURE 1:**
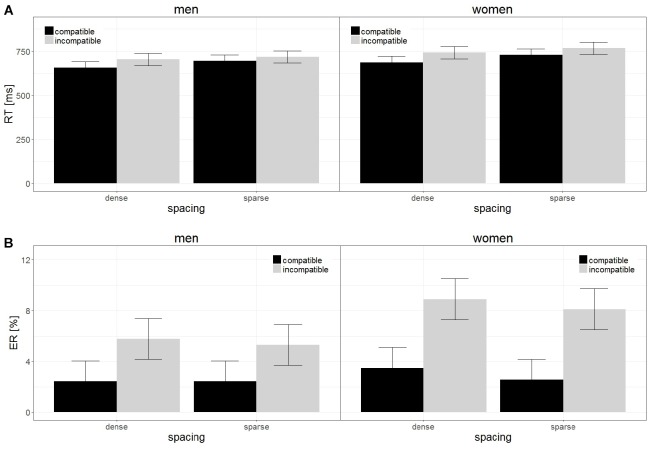
Reaction times **(A)** and Error rates **(B)** in the number comparison tasks. Participants took significantly longer and made more errors when responding to incompatible items compared to compatible items. This unit-decade compatibility effect was larger in women compared to men for error rates. The compatibility effect in reaction times was larger in sparsely spaced items compared to densely spaced items. Error bars represent standard errors.

In the analysis of RT, only spacing remained a significant effect in the model [*b* = −0.25, *SE*_b_ = 0.03, *t*_(248)_ = −7.19, *p* < 0.001]. Reactions were faster with dense (696.55 ms) compared to sparse spacing (726 ms). The main effect of sex and the interaction between sex and spacing were non-significant and thus removed from the model. Results are displayed in Figure 1A.

#### Error Rates

In the analysis of ER, only sex remained a significant effect in the model [*b* = 0.30, *SE*_b_ = 0.15, *t*_(81)_ = 2.10, *p* = 0.04]. Women made on average more errors (5.76%) than men (3.99%). Figure 1B shows that this difference is attributable to incompatible rather than compatible items. The main effect of spacing and the interaction between sex and spacing were non-significant and thus removed from the model.

### Effect of Sex and Spacing on the Compatibility Effect

For both, the compatibility effect in RT and ER, linear mixed models were performed including participant number as a random factor and spacing (dense vs. sparse), as well as sex (men vs. women) and their interactions as fixed effects. Age and IQ were entered as covariates. Neither age nor IQ had a significant effect on the compatibility effect in RT or ER. These variables were thus removed from the models. Table 1 summarizes the respective zero-order correlations.

#### Reaction Times

In the analysis of RT, only spacing remained a significant effect in the model [*b* = 0.47, *SE*_b_ = 0.15, *t*_(82)_ = 3.24, *p* = 0.002], indicating a larger compatibility effect in RT with dense (50.58 ms) compared to sparse spacing (30.57 ms). The main effect of sex and the interaction between sex and spacing were non-significant and thus removed from the model. Results are displayed in Figure 1A.

#### Error Rates

In the analysis of ER, only sex remained a significant effect in the model [*b* = 0.36, *SE*_b_ = 0.18, *t*_(81)_ = 2.04, *p* = 0.04], indicating a larger compatibility effect in ER in women (5.49%) compared to men (3.10%). The main effect of spacing and the interaction between sex and spacing were non-significant and thus removed from the model. Results are displayed in Figure 1B.

### Effect of Sex Hormones on the Compatibility Effect

Mean hormone values are displayed in Table 2. Testosterone was significantly higher in men compared to women [*t*_(77)_ = 6.54, *p* < 0.001], while progesterone was significantly higher in women compared to men [*t*_(76)_ = −5.40, *p* < 0.001]. Estradiol was by trend higher in women compared to men [*t*_(76)_ = −1.94, *p* = 0.06].

**Table 2:** Sex hormone levels in men and women.

	Men [Mean ± SD]	Women [Mean ± SD]
Testosterone [pg/ml]	117.99 ± 47.35	62.93 ± 24.09
Estradiol [pg/ml]	2.83 ± 1.64	3.60 ± 1.88
Progesterone [pg/ml]	84.54 ± 57.89	190.90 ± 107.38

To evaluate whether sex hormones mediated the sex differences in the compatibility effect in ER, mediation analyses were performed using estradiol, progesterone and testosterone as well as the progesterone/testosterone ratio as potential mediators. In a first step, hormone values were entered as predictors in linear mixed models including participant number as a random factor and the compatibility effect in ER as dependent variable. Neither estradiol nor progesterone nor testosterone showed a significant influence on the compatibility effect in ER [estradiol: *b* = 0.01, *SE*_b_ = 0.10, *t*_(76)_ = 0.12, *p* = 0.91; progesterone: *b* = 0.15, *SE*_b_ = 0.10, *t*_(76)_ = 1.63, *b* = 0.11; testosterone: *b* = −0.13, *SE*_b_ = 0.10, *t*_(77)_ = −1.40, *p* = 0.17]. They were thus not considered further as mediators. Table 1 shows that sex hormones were also not related to the compatibility effect in RT and only progesterone relates to overall RT and ER.

However, the progesterone/testosterone ratio was a significant positive predictor for the compatibility effect in RT [*b* = 0.22, *SE*_b_ = 0.09, *t*_(76)_ = 2.32, *p* = 0.02]. Participants with higher progesterone and lower testosterone levels had a larger compatibility effect in ER. Mediation analyses revealed that while there was a significant total effect of sex and the progesterone/testosterone ratio (*b* = 0.45, *SE*_b_ = 0.28, *p* < 0.001), neither the direct nor the indirect effect reached significance (ADE: *b* = 0.27, *SE*_b_ = 0.36, *p* = 0.14; ACME: *b* = 0.17, *SE*_b_ = 0.22, *p* = 0.20).

### Effect of Navigation Strategy on the Compatibility Effect

In order to test the hypothesis that participants with a stronger focus on landmarks show a higher compatibility effect, we performed a linear mixed effects model on the compatibility effect in ER using participant number as a random factor and sex, the strategy effect in navigation time as well as their interaction as independent variables. The final model included the strategy effect as a significant negative predictor [*b* = −0.23, *SE*_b_ = 0.09, *t*_(80)_ = −2.60, *p* = 0.01]. Participants with a smaller strategy effect, i.e., better performance with landmark trials during spatial navigation showed a larger compatibility effect. The main effects of sex and the sex^∗^strategy interaction did not reach significance and where thus removed from the model. This suggests that the effect of navigation strategy on the compatibility effect was comparable between men and women and that navigation strategy mediates the sex difference in the compatibility effect. Indeed a mediation analysis confirms a significant causal mediation of sex differences in the compatibility effect by navigation strategy (ADE: *b* = 0.31, *SE*_b_ = 0.29, *p* = 0.10; ACME: *b* = 0.08, *SE*_b_ = 0.06, *p* = 0.02).

## Discussion

This study set out to investigate whether sex differences in the compatibility effect during number comparison (i) depend on situational aspects like the vertical spacing between the two numbers to be compared, (ii) are mediated via sex hormone levels, and (iii) relate to sex differences in spatial navigation strategies. We expected to replicate a larger compatibility effect in women compared to men and with dense vertical spacing compared to sparse vertical spacing. Regarding the first question, we hypothesized a significant interaction between sex and vertical spacing in such a way that the sex difference in the compatibility effect is larger with larger vertical spacing between the numbers. Regarding the second question, we hypothesized the compatibility effect to relate positively to progesterone and negatively to testosterone. Regarding the third question, we expected the compatibility effect during number comparison to relate to landmark-based strategies during spatial navigation.

Regarding the first hypothesis, we were able to confirm a larger compatibility effect in women compared to men for ER, while the sex difference in the compatibility effect in RT did not reach significance. This is in line with previous results from a large scale online study (Huber et al., 2017), but note that most previous studies observe sex differences in the compatibility effect in RT, but not in ER (Pletzer et al., 2013; Harris et al., 2018). One possible explanation for this discrepancy is that in the present study, no overall reaction time differences were observed between men and women. When speed is matched between groups, differences are likely to manifest in ER. Notably, like in the study of Huber et al. (2017), sex differences in the compatibility effect were not explained by age or IQ. In summary, these results support the notion of a stronger individual tendency to process multi-digit numbers in a decomposed manner in women compared to men. The results are however not in line with the central presentation condition of our previous behavioral study (Harris et al., 2018). Note however that a sex difference in the compatibility effect was observed in that study, when stimuli were presented to the left and right hemifield, respectively. The inconsistencies between those two studies were the reason for the hypotheses that sex differences in the compatibility effect may depend on task characteristics like the vertical spacing between the numbers to compare.

Indeed, we were able to confirm a larger compatibility effect in RT for dense as compared to sparse vertical spacing in both women and men, as we had previously demonstrated for men (Pletzer et al., 2016). However, contrary to our hypothesis, no interaction between sex and vertical spacing was observed in either RT or ER. Thus, sex differences and stimulus characteristics seem to affect the compatibility effect separately, but not interactively. While it is possible that power considerations may have prevented us from detecting this interaction, the results suggest that the inconsistencies between our previous studies cannot be attributed to the differences in the vertical spacing of numbers. Rather, other factors may have contributed to these inconsistencies.

For instance, no sex differences in overall performance were observed in the neuroimaging study (Pletzer et al., 2013), but in the behavioral study (Harris et al., 2018). In the present study again, no difference in overall speed, but in accuracy was observed. It is thus possible, that a sex difference in the compatibility effect is harder to detect if one group starts out with lower performance due to a ceiling effect. Thus, the inconsistencies between the two previous studies may simply be the result of a sampling bias. Indeed, both previous studies did not explicitly control for a matched IQ between men and women, but implicitly assumed similar IQ based on similar education and social background. It is possible that this assumption was not met in the Harris et al. (2018) study. In the present study we did explicitly match IQ between the male and female group, which resulted in matched speed between men and women. Such a setting is more adequate to detect differences in processing strategies between groups.

Since a significant sex difference was only observed on the compatibility effect in ER, the following discussion refers to the compatibility effect in ER.

Regarding the second hypothesis, absolute sex hormone levels were not related to the compatibility effect. However, the progesterone/testosterone ratio related positively to the compatibility effect, which is in line with the assumption that participants with higher progesterone, but lower testosterone levels show a larger compatibility effect. While the effect was rather small, it is in line with previous observations that progesterone relates to local and testosterone to global processing (Pletzer et al., 2014). However, the progesterone/testosterone ratio did not explain the sex difference in the compatibility effect, suggesting other contributing factors.

Regarding the third hypothesis, the strategy effect in spatial navigation was indeed related to the compatibility effect during number comparison. The better participants performed with landmark-based instructions in the navigation task, the larger was their compatibility effect. We were previously able to demonstrate better performance with landmark-based instructions in women compared to men (Harris et al., unpublished). Unlike the progesterone/testosterone ratio, navigation strategy did explain the sex difference in the compatibility effect in a mediation analysis. While both spatial and numerical processing strategies have previously been related to global-local processing (Pletzer et al., 2017; Pletzer et al., unpublished), this is the first report linking holistic vs. decomposed processing strategies across the spatial and numerical domain.

In summary the present study corroborates previous findings that both sex and stimulus characteristics influence the compatibility effect during number magnitude processing. However, our data suggest that these factors contribute separately, but not interactively to the tendency of processing multi-digit numbers in a holistic or decomposed manner. Furthermore, our data suggest that sex differences in the compatibility effect cannot be explained by sex hormone levels, but by spatial processing strategies. This is the first report linking sex differences in number magnitude processing to sex differences in spatial processing.

## Author Contributions

BP designed the study, performed the analyses and wrote the manuscript. TH and AS assisted in data collection.

## Conflict of Interest Statement

The authors declare that the research was conducted in the absence of any commercial or financial relationships that could be construed as a potential conflict of interest.

## Footnotes

1 https://online.uni-salzburg.at/plus_online/wbMitteilungsblaetter.display?pNr=98160
